# Sternalis muscle revisted in South Indian male cadaver: a case report

**DOI:** 10.4076/1757-1626-2-6318

**Published:** 2009-07-30

**Authors:** Kumar MR Bhat, Bhagath Kumar Potu, Siddaraju Gowda

**Affiliations:** Department of Anatomy, Centre for Basic Sciences, Kasturba Medical College, Manipal UniversityManipal, KarnatakaIndia-576104

## Abstract

**Introduction:**

Sternalis/rectus sternalis is a rare muscle found in the chest wall. Only 3-5% of the cases are found world wide.

**Case presentation:**

Here we report the case of Sternalis in the 60-year-old South Indian male cadaver. This report discusses the origin, orientation, relations of this muscle.

**Conclusion:**

Importance of the knowledge of the presence of these rare muscles in clinical diagnosis and therapeutic implications is discussed.

## Introduction

The sternalis muscle is a small supernumerary muscle located in the anterior thoracic region, superficial to the sternum and the sternocostal fascicles of the pectoralis major muscle. Turner named it for the first time in his book *Anatomes Elenchus Accuratissimus* published in 1604 [1] and identified precisely in 1726 by Du Puy. This muscle has been reported in both males and females, and with a variable frequency in different ethnic groups [2]. Its incidence is low, approximately 3-5% [3]; a whole year can easily go by without encountering a sternalis muscle in the dissecting room [4]. According to Costa [5], it was termed “abdomino-guttural” by Duges; “abdomino-cutaneous” by Klein; “sternalis brutorum” by Kuhff; and “cutaneous pectoris” by Zenker.

Although sternalis muscle was first described 3 centuries ago, its origin is still unclear. Some authors suggest that it originates from adjacent muscles such as the sternocleidomastoid muscle, pectoralis major muscle, rectus abdominis muscle, remnant of panniculus carnosus etc. Novakov and his co-worker describe it as derived from pectoralis major muscle [6]. Here we describe a case of the sternalis muscle observed unilaterally in a cadaver.

## Case presentation

**Figure 1.**
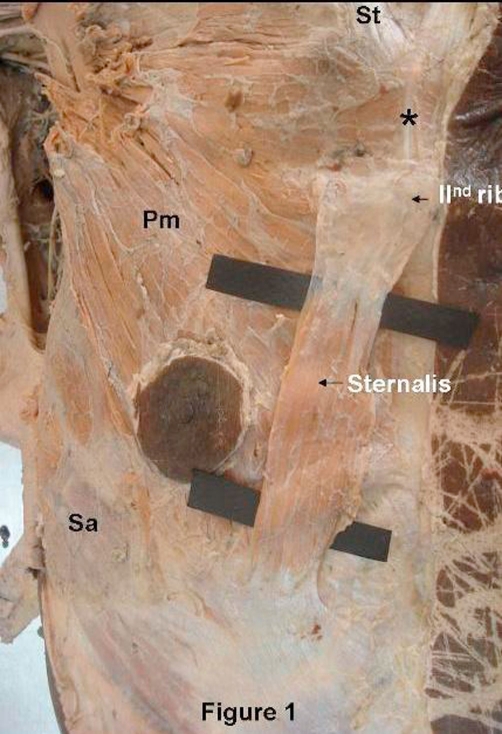
Thin strip of Sternalis muscle, obliquely arranged superficial to left Pectoralis major muscle. Aponeurotic extension between Sternalis and sternocledomastoid muscles.

During dissection of the thoraco-abdominal region of a 60-year-old male, we observed a distinct muscular mass about 12.3 cm long in the left hemithorax, covered by superficial fascia and located anterior obliquely to the pectoralis major muscle. Its cranial part is blends as aponeurosis with fibers of the sternochondrocostal portion of the left pectoralis major muscle close to second costo-chondral junction. Then, few aponerotic fibers extended to blend with sternal origin of sternocledomastoid muscle and sternoclavicular joint. Its caudal end blends as aponeurosis with fascia covering pectoralis major, aponeurosis of external oblique muscle of the abdomen and lower segments of the serratus anterior muscle, 4 cm lateral to midsternal line at the level of 7^th^ to 8^th^ costal cartilages (Figure 1).

## Discussion

Recent reports by Scott-Conner and Al-Jurf [7] and Bailey and Tzarnas [8] indicate that despite numerous descriptions of the sternalis muscle in the literature, the muscle is relatively unknown by clinicians. These authors pointed out that the discussion of the muscle is non-existent during medical training or seldom included in standard medical texts. Ruge [9] considers the sternalis to represent a vestige of the cuticular muscle of mammals that constitutes the great subcutaneous muscle of the trunk; it also presents in man in the form of the axillary arch. Barlow [10] claims that the sternalis muscle represents the remains of the panniculus carnosus. It has been considered that, sternalis to be a variant of pectoralis major [11] and Sadler [12] describes it as part of a ventral, longitudinal column of muscle arising at the ventral tips of the hypomeres. In general, when a muscle contracts the insertion is pulled toward its origin. Therefore, because of its particular location, it has been suggested that contraction of the sternalis muscle can elevate the lower part of the chest. Thus, sternalis plays only an accessory role in this function [13]. Its presence may suggest a diagnosis of hernia of the pectoralis muscle to the examining physician [14]. According to a recent review by Bradley et al. [15], the sternalis muscle is identified in only four of approximately 32,000 patients during mammography screening. The sternalis can present alterations on the ECG [16] or occasionally be wrongly interpreted as a mass requiring surgical resection [15]. Presence of rectus sternalis may interfere with the submuscular pocket dissection when an intraalveolar or submammary approach is used and it can be used to cover the prosthesis in its most medial part [17] and can be used for reconstruction surgery after mastectomy [18].

This rare anomaly has puzzled radiologists and surgeons in confirming diagnosis or mistaking it for a tumor on mammography or CT scan. Therefore, knowledge of this rare variation is not only of interest to morphologists, but also important for clinicians. Its location, direction and thickness are essential for general and plastic surgeons as well as for the specialists in imaging diagnostics.

## Abbreviations

Pm: Pectoralis major muscle
Sa: Serratus anterior muscle
St: Sternocledomastoid muscle
